# Expression of the inhibitor of DNA-binding (ID)-1 protein as an angiogenic mediator in tumour advancement of uterine cervical cancers

**DOI:** 10.1038/sj.bjc.6604722

**Published:** 2008-10-28

**Authors:** M K Maw, J Fujimoto, T Tamaya

**Affiliations:** 1 Department of Obstetrics and Gynecology, Graduate School of Medicine, Gifu University School of Medicine, 1-1 Yanagido, Gifu City 501-1194, Japan

**Keywords:** ID-1, uterine cervical cancer, angiogenesis, prognostic indicator, tumour advancement

## Abstract

The ID protein, an inhibitor of basic helix-loop-helix (HLH) transcription factors, has been involved in multiple cellular processes. To investigate the association between tumour advancement and ID expressions of uterine cervical cancers, the levels of ID-1, ID-2 and ID-3 mRNAs were determined by real-time reverse transcription-polymerase chain reaction and the histoscore with the localisation of ID-1 was determined by immunohistochemistry and patient survival in 60 patients. ID-1 histoscores and mRNA levels both significantly (*P*<0.05) increased in uterine cervical cancers according to clinical stage regardless of histopathological type or lymph node metastasis. Furthermore, the 36-month survival rate of the 30 patients with high ID-1 was poor (60%), whereas that of the other 30 patients with low ID-1 was significantly higher (83%). ID-1 histoscores and mRNA levels significantly (*P*<0.0001) correlated with microvessel counts in uterine cervical cancers. Tumour cells show mostly diffuse to strong cytoplasmic expression of ID-1 and also very faint expression in endothelial cells. Moreover, ID-1 expression not only correlated with microvessel counts but also correlated significantly with histoscore. Therefore, ID-1 might work on tumour advancement through angiogenic activity and is considered to be a candidate for a prognostic indicator in uterine cervical cancers.

The family of inhibitors of DNA-binding (ID) proteins consists of four members of basic helix-loop-helix (bHLH) transcription factors lacking the DNA-binding domain (Norton *et al*, 1998a). Therefore, they act as dominant-negative regulators of bHLH proteins by forming transcriptionally inactive Id-bHLH protein complexes (Riechmann *et al*, 1994; Riechmann and Sablitzky, 1995). ID proteins play key roles in the regulation of cell differentiation during neurogenesis, lymphoiesis and angiogenesis. Their functions also include the promotion of cell growth and cell cycle progression, and apoptotic induction (Lyden *et al*, 1999; Norton, 2000).

Regarding tumour cell proliferation, ID-1 inhibited the promoter region of the tumour suppressor gene CDKN2A/p16, supporting the role of ID-1 as a potential oncogene (Alani *et al*, 2001). Furthermore, ID proteins also inhibited the transformation-specific sequence (ETS) transcription factors of an avian erythroblastosis virus, E26 (Yates *et al*, 1999), which are able to suppress p16 expression by binding to and activating its promoter. This indicates that IDs might inhibit the p16 promoter, and thereby increase tumour cell proliferation, through interactions with ETS transcription factors directly and indirectly (Ohtani *et al*, 2001). Both IDs and ETS transcription factors have previously been implicated in the regulation of angiogenesis (Lyden *et al*, 1999; Wernert *et al*, 2003). The ID interactors–E protein transcription factors E12 (also known as epididymal sperm-binding protein, ELSPBP1), E47 (also known as E2A and TCF3), E2-2 (also known as TCF4) and HEB (also known as TCF12) (Yan *et al*, 1997), retinoblastoma protein (RB) (Lasorella *et al*, 2000) and ETS proteins (Ohtani *et al*, 2001)–are all implicated in tumorigenesis.

Recently, several reports have shown that ID-1 protein can induce cell proliferation and increase DNA synthesis, and that it can immortalise mammalian cells in corporation with other oncogenes (Norton and Atherton, 1998b; Alani *et al*, 1999). ID-1 overexpression has been found in several types of primary human cancers including breast, pancreatic, prostate, nasopharyngeal, melanoma and cervical cancers (Langlands *et al*, 2000; Lin *et al*, 2000; Polsky *et al*, 2001; Schindl *et al*, 2001, 2003; Wang *et al*, 2002; Ouyang *et al*, 2002a; Schoppmann *et al*, 2003; Lee *et al*, 2004; Straume and Akslen, 2005; Jang *et al*, 2006). IDs activate VEGF-dependent mobilisation of circulating endothelial cells and endothelial precursor cells from the bone marrow (Lyden *et al*, 2001). Further, ID-1 might act by transcriptional repression of thrombospondin (TSP)-1, a well-known angiogenesis inhibitor (Volpert *et al*, 2002). TSP-1 was more frequently expressed in leiomyoma compared with uterine smooth muscle tumour of uncertain malignant potential and leiomyosarcoma (Bodner-Adler *et al*, 2006).

Previous studies reported, expression of ID-1 was shown as an independent prognostic factor in cervical cancer with long-time follow-up, even more significant in early-stage disease. Over expression of ID-1 is associated with more aggressive behavior of tumour cells in cervical cancer (Schindl *et al*, 2001). However, no evidence clarifying the direct association between tumour advancement and ID protein expression is available in human primary cancer so far.

To elucidate the clinical implication of IDs in uterine cervical cancers, we analysed the manner expression of ID proteins in uterine cervical cancer progression according to clinical backgrounds, their influence on the survival of the patients and the association between ID mRNA expression and neoangiogenesis, assessed by assessment of microvessel density (MVD).

## Materials and methods

### Patients and tissues

Prior informed consent for the following studies was obtained from all patients and approval was given by the Research Committee for Human Subjects, Gifu University School of Medicine. Sixty patients ranging from 35 to 79 years of age with uterine cervical cancers (stage I, 21 cases; stage II, 21 cases; and stage III, 18 cases; squamous cell carcinoma (SCC), 42 cases and adenocarcinoma (AD), 18 cases) underwent surgery at the Department of Obstetrics and Gynecology, Gifu University School of Medicine, between September 1998 and August 2004. Patient prognosis was analysed in relation to a 36-month survival rate. None of the patients had received any pre-operative therapy before the uterine cervical cancer tissue was taken in surgery. A part of each tissue of uterine cervical cancers was snap-frozen in liquid nitrogen and stored at −80°C to determine ID-1, ID-2 and ID-3 mRNA levels and those for immunohistochemistry were fixed with 10% formalin and embedded in paraffin wax. The clinical stage of uterine cervical cancers was determined by International Federation of Obstetrics and Gynecology (FIGO) classification (International Federation of Obstetrics and Gynecology (FIGO) News, 1989).

### Immunohistochemistry

Sections (4 *μ*m) of formalin-fixed paraffin-embedded tissue samples from uterine cervical cancers were cut with a microtome and dried overnight at 37°C on a silanised-slide (Dako, Carpinteria, CA, USA). The protocol of universal Dako-labelled Streptavidin–Biotin kit (Dako, Carpinteria, CA, USA) was followed for each sample. Samples were deparaffinised in xylene at room temperature for 30 min, rehydrated with graded ethanol and washed in phosphate-buffered saline (PBS). The samples were then placed in 10 mM citrate buffer (pH 6.0) and boiled in a microwave for 10 min for epitope retrieval. Endogenous peroxidase activity was quenched by incubating tissue sections in 3% H_2_O_2_ for 10 min. The primary antibodies, rabbit anti-human ID-1 (SC-734, Santa Cruz Biotechnology Inc., Santa Cruz, CA, USA), mouse CD34 (Dako, Glostrup, Denmark) and rabbit anti-factor VIII-related antigen (Zymed, San Francisco, CA, USA) were used overnight at 4°C at dilutions of 1 : 50, 1 : 40 and 1 : 2, respectively. The slides were washed and biotinylated secondary antibody (Dako, Carpinteria, CA, USA) was applied for 30 min after rinsing in PBS, after which streptavidin-conjugated horseradish peroxidase (Dako, Carpinteria, CA, USA) was added for 30 min. Slides were then washed and treated with the chromogen 3,3′–diaminobenzidine (Dako, Carpinteria, CA, USA) for 5 min, then rinsed in PBS, and counterstained with Mayer's haematoxylin, dehydrated in graded ethanols, cleared in xylene and cover-slipped with a mounting medium, Entellan New (Merck, Darmstadt, Germany). For confirmation of the specificity for ID-1 antigen, we also used another ID-1 (SC-488) rabbit polyclonal antibody (Santa Cruz Biotechnology Inc., Santa Cruz, CA, USA) and we have observed the exact identified intensity and localisation of staining for ID-1 expression in tumour cells as ID-1 (SC-734) antibody. For the negative controls, the primary antibodies of ID-1, CD34 and factor VIII-related antigen were omitted and the corresponding preimmune animal serums (rabbit, mouse and rabbit, respectively) (Dako, Carpinteria, CA, USA) were used instead.

### Assessment of histochemical score (histoscore)

All sections of immunohistochemical staining for ID-1 were evaluated in a semiquantitative fashion according to the method described by McCarty *et al* (1985), which considers both the intensity and the percentage of cells stained in each of five intensity categories. Intensities were classified as 0 (no staining), 1 (weak staining), 2 (distinct staining), 3 (strong staining) and 4 (very strong staining). For each stained section, a value-designated histoscore was obtained by application of the following algorithm: histoscore=∑(*i*+1) × *Pi*, where *i and Pi* represent intensity and percentage of cells that stain at each intensity, respectively, and corresponding histoscores were calculated separately. Results were assigned to four groups according to their overall scores: weak, <160; distinct, 161<, >220; strong, 221<, >280; very strong, 280<. Immunohistochemistry results were analysed by two pathologists.

### Assessment of microvessel density

The MVD was assessed with microvessel counts (MVCs) in sequential tissue sections stained with mouse CD34 and rabbit factor VIII-related antigen antibodies. Blood vessels with a clearly defined lumen or a well defined linear vessel shape, but not single endothelial cells, were taken into account for microvessel counting (Giatromanolaki *et al*, 2001). Fives areas of highest vascular density were chosen and microvessel counting was performed at × 200 magnification by two investigators. The MVCs were determined as the mean of the vessel counts obtained from these fields (Maeda *et al*, 1996).

### Preparation of standard template for real-time reverse transcription–polymerase chain reaction (RT–PCR)

Internal standard template for real-time PCR was produced by PCR amplification using the primers of ID-1 gene, 418–782 in the cDNA (ID-1-TS: 5′-TTGGAGCTGAACTCGGAA-3′ and ID-1-TAS: 5′-TCTCTGGTGACTAGTAGGT-3′); ID-2 gene, 907–1253 in the cDNA (ID-2-TS: 5′-CTAAGCAGACTTTGCCTTT-3′ and ID-2-TAS: 5′-CTGAAATAAAGCAGGCAATC-3′); ID-3 gene, 686–1009 in the cDNA (ID-3-TS: 5′-GAACTTGTCATCTCCAACGA-3′ and ID-3-TAS: 5′-CACGCTCTGAAAAGACCT-3′). The DNA template was purified using a GeneClean II kit (Qbiogene, Irvine, CA, USA). The copy numbers of the standard template were determined to quantitate ID-1, ID-2 and ID-3 mRNA level in samples for real-time RT–PCR.

### Real-time RT–PCR to amplify ID-1, ID-2 and ID-3 mRNAs

Total RNA was extracted with the acid guanidinium thiocyanate–phenol–chloroform method (Chomczynski and Sacchi, 1987). The total RNA (3 *μ*g) was reverse transcribed using Moloney murine leukaemia virus reverse transcriptase (MMLV-RT, 200 U *μ*l^−1^, Invitrogen, Carlsbad, CA, USA) and the following reagents: 250 mM Tris-HCl, pH 8.3, 375 mM KCl, 15 mM MgCl_2_, 0.1 M dithiothreitol, 10 mM deoxynucleotide (deoxyadenosine, deoxythymidine, deoxyguanosine and deoxycystidine) tri-phosphates (dNTPs) mixture and random hexamers (Invitrogen) at 37°C for 1 h. The reaction mixture was heated for 5 min at 94°C to inactivate MMLV-RTase.

Real-time PCR was performed with a Takara Ex Taq R-PCR kit, Version 1.0 (Takara, Otsu, Japan), using a smart cycler system (Cepheid, Sunnyvale, CA, USA). The reaction solution (25 *μ*l) contained Takara Ex Taq HS (5 U *μ*l^−1^), 10 × R-PCR Buffer (Mg^2+^ free), 250 mM Mg^2+^ solution, 10 mM dNTP mixture, SYBR Green I (1 : 1000 dilution; CambrexBio Science, Rockland Inc., Rockland, ME, USA) and 20 *μ*M of the primers of ID-1 gene, 545–675 in the cDNA (ID-1-S: 5′-ACGATCGCATCTTGTGTC-3′ and ID-1-AS: 5′-CTTGTTCTCCCTCAGATCC-3′); ID-2 gene, 907–1026 in the cDNA (ID-2-S: 5′-CTAAGCAGACTTTGCCTTT-3′ and ID-2-AS: 5′-CATTCAGTAGGCTTGTGTC-3′); ID-3 gene, 709–873 in the cDNA (ID-3-S: 5′-AAGGAGCTTTTGCCACTGA-3′ and ID-3-AS:5′-CCAGGAAGGGATTTGGTGAA-3′) with the transcribed total RNA from the tissue and a serially diluted standard template. The real-time PCR reactions were initially denatured by heating at 95°C for 30 s, followed by 40 cycles consisting of denaturation at 94°C for 10 s, annealing at 55°C for 5 s and extension at 72°C for 20 s. A strong linear relationship between the threshold cycle and the log concentration of the starting DNA copy number was always shown (correlation coefficient >0.99). Quantitative analysis was performed to determine the copy number of each sample.

### Statistical analysis

Inhibitor of DNA-binding-1, ID-2 and ID-3 mRNA levels were determined from three parts taken from each tumour, and each sample was analysed in triplicate. ID-1 histoscores and mRNA levels were compared using Student's *t*-test. The 24-month survival rate was calculated according to the Kaplan–Meier method, and analysed with the log-rank test. The correlations between ID-1 histoscores and mRNA levels with MVCs were performed with bivariate Pearson's correlation. Differences were considered significant when *P* was less than 0.05.

## Results

**Figure 1:**
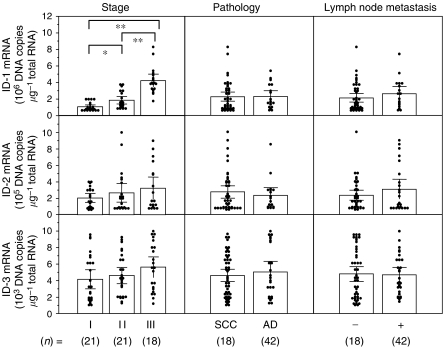
Inhibitor of DNA-binding-1, ID-2 and ID-3 mRNA levels in uterine cervical cancers classified according to clinical stage, histopathological type and lymph node metastasis. Clinical staging of uterine cervical cancers was done according to FIGO. Each level is the mean±s.d. of nine determinations. SCC, squamous cell carcinoma; AD, adenocarcinoma. ^*^P<0.05; ^**^P<0.001.

Inhibitor of DNA-binding-1 mRNA levels significantly increased with increasing clinical stages (I<II<III; *P*<0.05) of uterine cervical cancers, regardless of histopathological type and lymph node metastasis (Figure 1). However, there was no significant difference in ID-2 or ID-3 mRNA levels according to clinical stage, histopathological type or lymph node metastasis in uterine cervical cancers, as shown in Figure 1. These results prompted us to concentrate our investigation on ID-1 in uterine cervical cancers.

**Figure 2:**
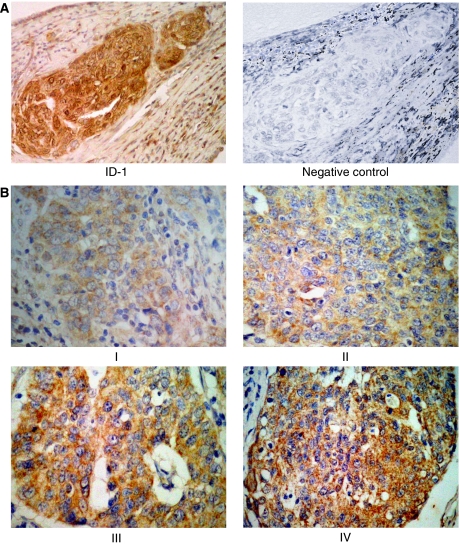
(**A**). Immunohistochemical staining for positive and negative ID-1 staining in squamous cell carcinoma of uterine cervical cancers. (**B**). Immunohistochemical staining for ID-1 representing cases from each stage in uterine cervical cancers. Weak staining (score 111) stage I; distinct (score 167) stage II; strong (score 264) stage III and very strong (score 392) stage IV, showing strong cytoplasmic expression in tumour cells. Overall scores: weak, <160; distinct, 161<, >220; strong, 221<, >280; very strong, 280<. (original magnification × 400). Rabbit anti-human ID-1 (SC-734, Santa Cruz Biotechnology Inc., Santa Cruz, CA, USA) was used at a dilution of 1 : 50 as the primary antibody (original magnification × 200).

Inhibitor of DNA-binding-1 staining was diffusely located in the cancer cells. Although ID-1 expression in stroma cells was weak or negative, perivascular cells of intratumoral microvessels were strongly positive in all cases (Figure 2A, a representative case, 46-year-old patient, stage IIb, squamous cell carcinoma). Because ID-1 is not a transcription factor *per se*, it lacks the nuclear localisation signal found on many basic HLH proteins but gives a cytoplasmic signal instead (Maruyama *et al*, 1999; Lin *et al*, 2000; Schindl *et al*, 2001). ID-1 diffuse cytoplasmic staining was seen from moderate to strong intensity in most cases whereas nuclear staining was observed only occasionally (Figure 2B).

**Figure 3:**
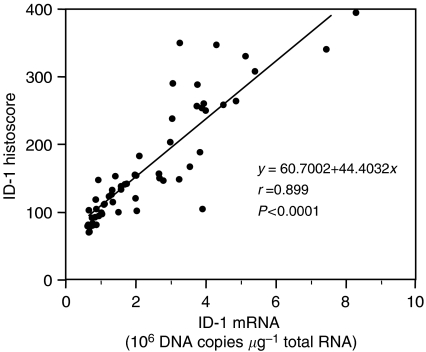
Correlation between ID-1 histoscores in cancer cells and mRNA (10^6^ DNA copies *μ*g^−1^ total RNA) levels in uterine cervical cancers. ID-1 histoscores and mRNA levels were determined by immunohistochemistry and real-time RT–PCR, respectively. Each level is the mean±s.d. of nine determinations.

**Figure 4:**
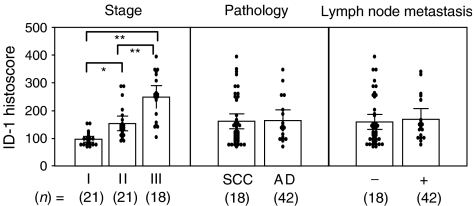
Inhibitor of DNA-binding-1 histoscores in uterine cervical cancers classified according to clinical stage, histopathological type and lymph node metastasis. Clinical staging of uterine cervical cancers was done according to FIGO. Each level is the mean±s.d. of nine determinations. SCC, squamous cell carcinoma; AD, adenocarcinoma. ^*^P<0.05; ^**^P<0.001.

Inhibitor of DNA-binding-1 histoscore in cancer cells significantly (*P*<0.001) correlated with corresponding mRNA levels in each tissue, as shown in Figure 3. Although there was no significant difference in ID-1 histoscores in cancer cells according to histopathological type and lymph node metastasis, ID-1 histoscores significantly (I<II<III; *P*<0.05) increased with increased clinical stages of uterine cervical cancers (Figure 4), as did ID-1 mRNA.

**Figure 5:**
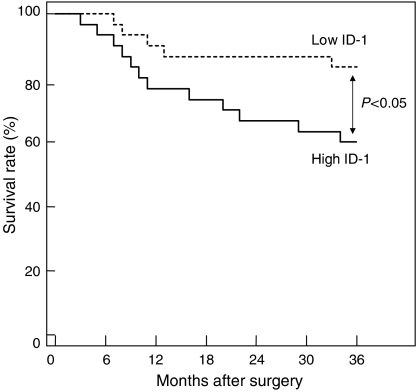
Survival rate after surgery for uterine cervical cancers. Patient prognosis was analysed with a 36-month survival rate. High ID-1, cases with high histoscores and mRNA levels (>140 histoscore and >1.6 × 10^6^ copies *μ*g^−1^ total RNA, respectively); *n*=30. Low ID-1, cases with low histoscores and mRNA levels (<140 histoscore and <1.6 × 10^6^ copies *μ*g^−1^ total RNA, respectively); *n*=30.

Furthermore, the 60 patients who underwent surgery were arbitrarily divided into two equal groups based on ID-1 histoscores and mRNA levels, with the midpoint being a histoscore of 140 and mRNA of 1.6 × 10^6^ copies *μ*g^−1^ total RNA, respectively. The two groups, determined independently by the ID-1 histoscores and mRNA levels, consisted of exactly the same patients. The prognosis of the 30 patients with high ID-1 (>140 histoscore and >1.6 × 10^6^ copies *μ*g^−1^ total RNA) in uterine cervical cancers was poor (60%), whereas the 36-month survival rate of the other 30 patients with low ID-1 (<140 histoscore and <1.6 × 10^6^ copies *μ*g^−1^ total RNA) was higher (83%), as shown in Figure 5.

**Figure 6:**
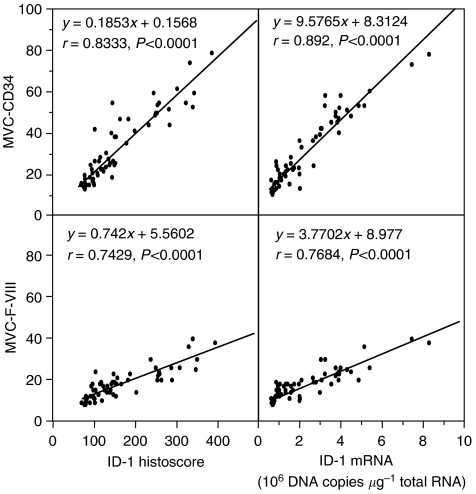
Correlation of ID-1 histoscores in cancer cells and mRNA levels in uterine cervical cancers with microvessel counts (MVCs). Each level is the mean±s.d. of nine determinations.

Inhibitor of DNA-binding-1 expression was very weak in endothelial cells although Factor VIII-related antigen and CD34 expression were strong. In tissue sections, weak ID-1 expression was associated with low MVD whereas strong ID-1 expression was associated with abundant microvessels. The ID-1 histoscores in cancer cells significantly correlated with MVC-CD34 (MVCs determined by immunohistochemistry for CD34; *r*=0.833, *P*<0.0001) and MVC-F-VIII (MVCs determined by immunohistochemistry for factor VIII-related antigen; *r*=0.742, *P*<0.0001); and ID-1 mRNA levels also correlated with MVC-CD34 (*r*=0.892, *P*<0.0001) and MVC-F-VIII (*r*=0.768, *P*<0.0001), as shown in Figure 6.

## Discussion

This study is, to our knowledge, the first to reveal that ID-1 expression in the cancer cells increased with tumour advancement of uterine cervical cancers. Also, the patients with high ID-1 expression had a lower survival rate compared with patients with low ID-1 expression. Negative prognostic impacts of increased ID-1 expression have been shown in breast cancers (Schoppmann *et al*, 2003), pancreatic cancers (Lee *et al*, 2004), melanoma (Straume and Akslen, 2005), cervical cancers (Schindl *et al*, 2001), and ovarian carcinomas (Schindl *et al*, 2003). This is in line with our finding. Therefore, ID-1 is recognised as a novel indicator of tumour advancement and patient prognosis in uterine cervical cancer.

Previously, no correlation between expression of ID proteins and angiogenesis, assessed by MVD was observed in cervical cancers (Schindl *et al*, 2001). But contrary to their finding, present study showed that mostly diffuse to strong cytoplasmic expression of ID-1 in tumour cells and also very weak ID-1 expression in endothelial cells. Moreover, ID-1 expression not only correlated with microvessel counts but also correlated significantly with histoscore. These data indicate that ID-1 overexpression is found to be closely related to tumour angiogenesis, a higher density of intratumoral vessel and poor survival in cervical cancer. Therefore, ID-1 is a candidate for angiogenic mediator as the clinical relevance of angiogenesis assessed by MVD.

Angiogenesis is essential for development, growth and advancement of solid tumours (Folkman, 1985). The angiogenic factors vascular endothelial growth factor (VEGF) (Fujimoto *et al*, 1999a) and thymidine phosphorylase identified with platelet-derived endothelial cell growth factor (Fujimoto *et al*, 1999b, 1999c, 2000) and interleukin (IL-8) along with the angiogenic transcriptional factor ETS-1 (Fujimoto *et al*, 2002) work on angiogenesis in uterine cancers.

Inhibitor of DNA-binding-1 is a regulator of basic HLH-mediated transcription (Norton *et al*, 1998a) and causes cells to pass a mitogen-restricted point in late G1 phase (Le Jossic *et al*, 1994). Therefore, ID-1 has been suggested as responsible for some of the changes in gene expression that lead to increased growth and invasion of tumour cells (Lyden *et al*, 1999). Moreover, the ectopic expression of ID-1 increases the proliferation, migration, invasion and metastasis of breast cancer cells (Desprez *et al*, 1998; Lin *et al*, 2000). Although ID-1 has been shown to interact with various cell cycle regulators (Alani *et al*, 2001; Ohtani *et al*, 2001), ID-1 seems to be an essential factor in the promotion of G1/S cell cycle transition in certain cancers by inactivating of p16 and increasing of CDK4 activity/RB (Polsky *et al*, 2001; Ouyang *et al*, 2002b; Singh *et al*, 2002). However, additional studies are required to determine the mechanism of interaction between ID-1 and various cell cycle regulators.

Recently, TSP-1, an endogenous inhibitor of neovascularisation has been identified as a target of ID-1 that is negatively regulated by ID-1 in the regulation of angiogenesis (Volpert *et al*, 2002). Thrombospondin-1 suppresses angiogenesis by inhibiting endothelial cell proliferation and inducing endothelial cell apoptosis (Zhang *et al*, 2003). For example, in the ID-1 −/− mice, transcription of TSP-1 is increased, which is associated with inhibition of bFGF and VEGF-induced angiogenesis. This phenotype can be reversed by inactivation of TSP-1 function (Volpert *et al*, 2002). In addition, microvessel density count was negatively associated with the intensity of TSP-1 expression in uterine cervical cancer (Wu *et al*, 2004). An inverse relation between ID-1 and TSP-1 expression supports the significant role of ID-1 in the regulation of this complex multitarget protein (Straume and Akslen, 2005). These previous studies suggested that due to ID-1 suppression, TSP-1 expression might be lost with tumour advancement, resulting in drastic tumour angiogenesis that accelerates tumour advancement. These provide evidence that clarifies the association between ID-1 overexpression and tumour angiogenesis in human primary cancer.

Inhibitor of DNA-binding-1 expression was high in two androgen-independent prostate cancer cell lines, DU145 and PC3, but its expression is undetectable in the androgen-dependent cell line LNCaP under serum-free conditions (Ling *et al*, 2003). As prostate cancer progression and aggressiveness are judged by its response to androgen, these results support the role of ID-1 expression in tumour progression. Furthermore, ID-1 expression was higher in highly aggressive breast cancer cell lines such as MDA-MB321 and MDA-MB-435 compared with the less aggressive cell lines such as MCF7 and T47D. Ectopic ID-1 expression in T47D cells led to increased cell growth, invasion ability and reduced sensitivity to sex hormones (Lin *et al*, 2000). In addition, stable transfection of breast cancer cells with the antisense ID-1 vector also reduces lung metastasis after their inoculation into nude mice (Fong *et al*, 2003). These results support the role of ID-1 in metastasis of breast cancer and also suggest inactivation of ID-1 as a therapeutic strategy in the treatment of metastatic breast cancer. Therefore, ID-1 protein may be an important new target molecule for antiangiogenic drug design in cancer treatment. It is strongly suggested that transcriptional suppression of TSP-1 by ID-1 may play an important part in promoting angiogenesis in the uterine cervical cancers.

To conclude, this study suggests that ID-1 promotes tumour advancement of uterine cervical cancer through angiogenesis, leading to poor prognosis, and provides a novel therapeutic approach in uterine cervical cancers.
