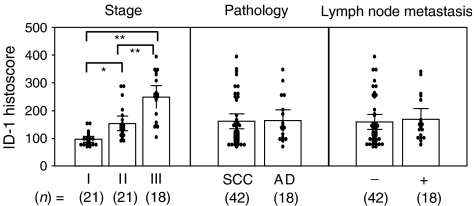# Expression of the inhibitor of DNA-binding (ID)-1 protein as an angiogenic mediator in tumour advancement of uterine cervical cancers

**DOI:** 10.1038/sj.bjc.6605205

**Published:** 2009-07-14

**Authors:** M K Maw, J Fujimoto, T Tamaya

**Correction to**: *British Journal of Cancer* (2009) **99,** 1557–1563, doi: 10.1038/sj.bjc.6604722

During proofing of the above paper, a number of mistakes were missed.

In the ‘Materials and Methods’ section, final paragraph (entitled ‘Statistical analysis’), the sentence beginning ‘The 24 month survival rate was calculated…’ should actually read ‘The 36-month survival rate was calculated…’

In Figure 1, the x axis labelling of the Pathology and Lymph node metastasis columns was incorrect – the correct Figure and labelling is shown below:

In Figure 4, the x axis labelling of the Pathology and Lymph node metastasis columns was again incorrect – the correct Figure and labelling is shown below:

## Figures and Tables

**Figure 1:**
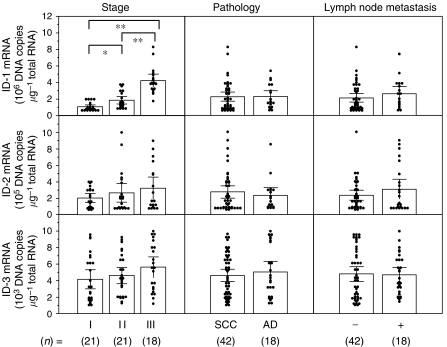


**Figure 4:**